# Health-Related Quality of Life in Patients with Primary Cutaneous Amyloidosis

**DOI:** 10.1371/journal.pone.0120623

**Published:** 2015-03-23

**Authors:** Sheng Fang, Xiao Shen, Ai-Jun Chen, Shuang Li, Kui Shan

**Affiliations:** 1 Department of Dermatology, The First Affiliated Hospital of Chongqing Medical University, Chongqing, China; 2 Department of burn and plastic surgery, The First Affiliated Hospital of Chongqing Medical University, Chongqing, China; Sickle Cell Unit, JAMAICA

## Abstract

**Background:**

Primary cutaneous amyloidosis (PCA) is a relatively rare and itchy skin disorder characterized by amyloid deposits in the superficial dermis. The cosmetic disfigurement and severe pruritus dramatically affects the patient’s quality of life. In spite of the prevalence of the disease in China, the quality of life (QoL) impact of the PCA has not been well defined and is the focus of this study.

**Objective:**

To examine the HRQoL of patients with PCA and to evaluate the association between HRQoL scores, disease, and socio-demographic determinants.

**Methods:**

A total of 104 PCA patients and 101 healthy participants completed the questionnaires. HRQoL was measured using dermatology life quality index (DLQI) and SF-36. The socio demographic and clinical data such as age, sex, duration of disease and distribution of lesion pattern were analyzed mainly by hierarchical multiple regression analyses.

**Results:**

Patients with PCA experienced significantly impaired health-related quality of life. The mean DLQI score was 9.05. Younger age, female gender, more pruritus and distribution pattern were independent predictor correlates of the high DLQI scores. The PCA group showed significantly decreasing average scores in several aspects of psychological symptoms, including SF, RE and MH.

**Conclusions:**

PCA disease has a negative impact on the HRQoL of patients, and the HRQoL is associated with various disease characteristics. In conjunction with medical interventions, psychological and sociocultural assessment and intervention should be an essential part of the management of these cases.

## Introduction

Primary cutaneous amyloidosis (PCA) is a relatively rare and itchy skin disorder characterized by amyloid deposits in the superficial dermis. PCA is defined as localized amyloidosis of the skin without evidence of systemic involvement. It usually includes 3 clinical types: lichen amyloidosis, macular amyloidosis and nodular amyloidosis [1]. Itching is a key characteristic of PCA that dramatically affects a patient’s quality of life (QoL). Cosmetic disfigurement can also cause patients to experience a different level of stigmatization, which can lead to psychosocial stress and the impairment of emotional functioning. Common dermatological disorders, such as vitiligo and psoriasis, can result in psychosocial effects and a low QoL. More than a cosmetic nuisance, skin diseases can produce anxiety, depression, and pruritus [2]. To assess the severity of PCA, QoL seems to be a more relevant criterion than clinical evaluation because the perception of patients may be different from doctors. Indeed QoL indicators are important in dermatology because skin diseases have a strong impact on social relations, daily activities and psychological health status [3–4]. This impact is more easily determined by the patient rather than by the physician. Although PCA was first described by Gutmann in 1928, it is seen most frequently in China and Southeast Asia [5]. In spite of the prevalence of the disease in China, the QoL impact of PCA has not been well defined and is the focus of this study. There have not been any studies assessing the QoL of patients with PCA. This study aimed to use the SF-36 dermatology life quality index (DLQI) to quantify the burden of PCA on the health-related QoL in Chinese PCA patients and attempted to identify the clinical and socio-demographic variables predicting the impairment of health-related quality of life in this cohort.

## Methods

### Patients

One hundred and four outpatients with PCA and one hundred and one comparison participants, ranging from 18–91 years in age, were included in the study. All the subjects were patients and diagnosed with PCA from September 2009 to October 2012 in the dermatology outpatient clinic at the affiliated hospital of Chongqing Medical University, Chongqing, China. A comparison group was recruited from healthy people who were undergoing routine health screening at the hospital. The screened subjects who were free of any significant medical illness by physical examination were invited to participate in the study. The healthy comparison group matched the study group for age, gender, educational level, residential locations, employment status and marital status. The Ethics Committee of the First affiliated hospital of Chongqing medical university granted permission to conduct this study. Written informed consent was obtained from each patient prior to the start of the study. The clinical diagnosis of PCA was confirmed by clinical features and histopathologic examination of biopsy material stained by crystal violet. Patients’ characteristics such as age, sex, age at onset of disease, duration of disease and distribution of lesion pattern were recorded.

### Instruments

A questionnaire used by the dermatologists to collect demographic and clinical information (age, gender, education, residential locations, current employment status and current marital status) was developed for all participants. PCA patients also answered questions regarding disease characteristics duration and involvement of different body sites. Pruritus severity was rated by the visual analogue scale (VAS), which was first developed in 1921 by Hayes & Patterson [6]. VAS is a graphic tool with a 100-mm horizontal line with the left end marked as “no symptom” and the right end marked as “worst imaginable symptom” [7]. Higher VAS scores mean more pruritus.

#### Dermatology life quality index

The DLQI, a simple, practical and self-administered questionnaire was designed to assess limitations caused by the impact of skin disease. It consists of ten items and covers six domains, namely symptoms and feelings, daily activities, leisure, work and school, personal relationships, and treatment. Each item with four possible answers scored from 0 to 3.The total score is calculated by summing the scores of all the items, resulting in a maximum score of 30 and a minimum score of 0. The scale scores are calculated for each domain, with higher scores representing a greater impact on quality of life. It has well-established reliability as well as content and provides validity in the dermatology setting. The Chinese version of this instrument was used in this study [8]. DLQI is an easy and efficient instrument for assessing the quality of life in patients with dermatological problems and offers good reliability and validity. Wang et al proved it could be used in both research and clinical settings in China. The internal consistency coefficient rates of this unidimensional measure were 0.87 (Cronbach’s alpha) and 0.85 (Spearman-brown’s), with high inter-correlations found between the dimensions and a correlation coefficient ranging from 0.4024–0.6569 [8].

#### Short form 36 health survey

The SF-36 is a 36-item general health status instrument often used in clinical trials and health services research. This is a self-administered questionnaire that contains 36 questions. The questions are summarized into eight scales: Physical Function (PF), Role Limitations-Physical (RP), Vitality (VT), General Health Perceptions (GH), Bodily Pain (BP), Social Function (SF), and Role Limitations: Emotional (RE) and Mental Health (MH). For each domain, a score ranging from 0 (worst measured health) to 100 (best measured health) was calculated. Previous studies have shown that the instrument discriminates well between perceptions of people with or without one or more chronic diseases with good reliability and validity. SF-36 has been validated for the general population in China. We used the Chinese version of the SF-36 [9].

### Statistical analysis

Statistical analysis was performed using the Statistical Package for the Social Sciences software, version 17 (SPSS, Chicago, IL, USA). Independent samples were analyzed, and t-tests were used to determine differences between different sample groups. Type one probabilities of <0.05 were considered statistically significant. The individual contribution of background variables on each outcome variable was analyzed by hierarchical multiple regression analysis. Demographic and disease related variables were first entered into the regression equation followed by the interactions of the demographic and disease related predictors.

## Results

### Study population characteristics

In total, 138 patients with PCA and 145 comparison participants were asked to complete the questionnaire. However, only 240 participants (120 patients and 120 comparison participants) responded to the request for participation. The response rate was 84.8%. Because of incomplete data and the paediatric patients, data from 104 patients with PCA and 101 from controls comparison were analyzed. The rate for exclusion was comparable in two groups: 16 (13.3%) in the PCA group and 19 (15.8%) in the control comparison group. The distribution of the participants was 104 PCA patients and 101 comparison patients. The study population characteristics are shown in Table 1. As presented in **Table 1**, 104 consecutive patients with PCA consisted of 43 male and 61 female patients, with a mean age of 43.7 years (range, 18–91). A total of 76 patients (73%) were married, and 59 patients (57%) were employed.

**Table 1:** Characteristics of recruited patients with PCA and comparison groups.

	Patients with PCA Total(n = 104)	Comparison group Total(n = 101)
Age (years),mean (range)	43.6(18–91)	42.3(18–87)
Gender, N (%)		
Male	43(41)	41(41)
Female	61 (59)	60 (59)
Age of onset (years),mean (range)	36.5(14–67)	
Duration of disease (years),mean (range)	5.3(1–33)	
Current marital status N (%)		
Married	76(73)	72(72)
Not married	28(27)	29(29)
Current employment status N (%)		
Employed	59(57)	65(65)
Unemployed	45(43)	36(36)
Skin affected pattern N (%)		
Cover	75(72)	
Uncover	21(20)	
Both	8(8)	

The comparison group consisted of 41 male and 60 female patients, with a mean age of 42.3 years (range, 18–87). A total of 72 patients (72%) were married, and 65 patients (65%) were employed. The duration of the disease was 5.3 (1–33) years, and the mean first onset age was 36.5 (14–67) years in the PCA group. Of the 104 participants in the PCA group, the skin lesions were present on uncovered parts (head, face, neck, forearm and hands, were considered as always uncovered) in 20% of patients, on covered parts (arms, legs, feet, trunk, genitals and other areas, were considered as always covered) in 72% of patients and on both types of parts in 8% of patients.

### Dermatology life quality index

The mean (± SD) DLQI score was high for PCA cases with a mean value of 9.05±3.88 of the total scores as well as the sub-scores **(Table 2).** Patients with PCA experienced significant impairment of life quality in terms of feelings, clothing, social and leisure activities and daily routine. The mean score was the highest for symptoms and feelings (2.29±1.05) and the lowest for work and school (0.98±0.73). The mean scores for other domains were as follows: personal relationships (1.67±0.77), daily activities (1.59±0.90), leisure (1.37±0.70), and treatment (1.15±0.68).

**Table 2:** DLQI scores in patients with PCA.

DLQI domains	Mean±SD	Minimum	Maximum
Symptoms feeling	2.29±1.05	0	5
Daily activities	1.59±0.90	0	4
Leisure	1.37±0.70	0	3
Work and school	0.98±0.73	0	3
Personal relationships	1.67±0.77	0	3
Treatment	1.15±0.68	0	3

### Hierarchical linear regression of DLQI onto demographic, clinical factors and interactions

With regard to the demographic and clinical variables, the multiple regression analysis identified that younger age, female gender, more pruritus and distribution pattern were independent predictor correlates of the high DLQI scores. The regression coefficients are shown in **Table 3.** Severity of pruritus (VAS scores) showed the strongest association with DLQI scores. The interaction term did not contribute significantly to the model except in the treatment scores. We found there was no significant association between the duration and DLQI scores except for the treatment scores.

**Table 3:** Hierarchical linear regression of DLQI onto demographic, clinical factors and interactions in PCA patients.

Variable	Symptoms and feeling		Daily activities		Leisure		Work and school		Personal relationship		Treatment		Total DLQI	
	Model 1	Model 2	Model 1	Model 2	Model 1	Model 2	Model 1	Model 2	Model 1	Model 2	Model 1	Model 2	Model 1	Model 2
Age	-0.175**	-0.267*	-0.172*	-0.104**	-0.220*	-0.077	-0.412**	-0.300	-0.211**	-0.328*	0.033	-0.078	-0.241**	-0.246
Gender^a^	0.457**	0.232	0.575**	0.780	0.282**	0.746**	0.206**	0.383*	0.349**	0.591**	0.322**	0.782**	0.472**	0.704**
Duration^b^	0.177	-0.403	0.036	0.221	0.057	0.251	0.050	0.384	-0.144	0.059	0.381**	-0.183	0.114	0.040
Distribution^c^	0.497**	0.304	0.503**	0.536*	0.382**	0.717**	0.378**	0.195	0.440**	0.054	0.342**	0.791**	0.539**	0.521**
VAS	0.595**	0.638*	0.337**	0.429*	0.475**	0.742**	0.407**	0.580*	0.459**	0.417	0.248**	0.398	0.536**	0.668
Interaction terms														
Age×duration		0.609*		-0.097		-0.144		-0.334		-0.168		0.689		0.141
Age×distribution		0.146		-0.005		-0.261		0.126		0.339		-0.412		0.010
Age×VAS		-0.145		-0.064		-0.078		-0.107		0.228		0.157		-0.016
Gender×duration		0.139		-0.211*		-0.171		-0.110		-0.113		-0.073		-0.098
Gender×distribution		0.069		-0.032		-0.116		0.087		0.076		-0.082		0.007
Gender×VAS		0.160		-0.077		-0.398*		-0.182		-0.266		-0.475**		-0.216
R^2^ change	0.754**	0.020	0.697**	0.017	0.454**	0.045	0.494**	0.021	0.608**	0.035	0.384**	0.106	0.782**	0.015
F change	59.966	1.372	45.608	0.909	16.282	1.393	19.160	0.679	30.378	1.520	12.199	3.188	70.442	1.110

Note: * P <0. 05, ** P<0. 01. ^a^ Men = 0, Women = 1. ^b^ In years. ^C^ cover = 0 uncover and both = 1. All the coefficients in the table are standardized regression coefficients

### Short form 36 health survey

The average individual scores on SF-36 for the two groups **(Table 4)** showed that the study group had significantly poorer scores than the comparison. The PCA group showed significantly decreasing average scores in several aspects of psychological symptoms, including SF, RE and MH. There were no significant differences in other aspects between the two groups, such as PF, RP, BP, GH and VT.

**Table 4:** Mean scores on SF-36 of the study group and the comparison group.

SF-36 Scales	PCA group(n = 104)	Comparison group (n = 101)	t	P
Physical Functioning (PF)	84.76±9.36	85.79±8.51	-0.826	0.410
Role Physical (RP)	65.86±18.45	68.32±18.67	-0.945	0.346
Body Pain (BP)	78.94±13.36	79.11±13.79	0.088	0.930
General Health (GH)	68.99±13.02	71.88±15.60	1.442	0.151
Vitality (VT)	64.38±13.79	67.33±16.64	1.381	0.169
Social Function (SF)	59.50±14.83	80.70±16.35	9.729	<0.001
Role Emotional (RE)	62.83±24.73	74.92±27.25	3.330	0.001
Mental Health (MH)	63.04±11.00	75.37±15.19	6.640	<0.001

### Hierarchical linear regression of SF-36 onto demographic, clinical factors and interactions

The results of hierarchical regression analysis on SF-36 are shown in **Table 5**. Women had a significantly poorer quality of life PF, SF and RE on the SF-36 scales. Younger patients had lower scores in several aspects except BP and GH. No significant differences in SF-36 scores, except for RP, were seen with different durations of the disease. Patients with severe pruritus showed a significantly more impaired quality of life than the others only for some emotional scales, such as SF, RE and MH. The distribution patterns of PCA also had a positive correlation with some aspects of emotional scales. The distribution pattern was also identified as an independent predictor correlate of the SF-36 scores. Patients with visible parts of lesions had lower scores on SF, RP, RE and MH. The interaction term did not contribute significantly to the model.

**Table 5:** Hierarchical linear regression of SF-36 on demographic, disease related variables and interactions in PCA patients.

Variable	Physical Function		Role Physical		Vitality		General Health		Bodily Pain		Social Function		Role Limitations—Emotional		Mental Health	
	Model 1	Model 2	Model 1	Model 2	Model 1	Model 2	Model 1	Model 2	Model 1	Model 2	Model 1	Model 2	Model 1	Model 2	Model 1	Model 2
Age	0.494**	0.223	0.359**	0.355	0.484**	0.115	-0.186	-0.307	-0.139	-0.106	0.234**	0.086	0.317**	0.257	0.092**	0.021
Gender^a^	-0.212**	0.069	-0.095	0.025	-0.015	0.508	0.004	-0.155	-0.090	-0.094	-0.360**	-0.379	-0.480**	-0.534	-0.030	-0.113
Duration^b^	-0.298	0.438	-0.203**	0.123	0.004	0.558	-0.136	0.070	-0.183	0.434	-0.050	-0.282	-0.112	0.007	-0.070	0.307
Distribution^c^	-0.097	-0.119	0.039	-0.237	0.045	0.130	0.063	-0.484	0.068	-0.517	-0.566**	-0.402	-0.382**	-0.378	-0.637**	-0.408
VAS	0.188	0.450*	-0.355**	-0.328	-0.007	0.581*	-0.088	-0.253	-0.155	-0.205	-0.563**	-0.768	-0.443**	-0.594	-0.614**	-0.981**
Interaction terms																
Age×duration		-0.778*		-0.390		-0.578		-0.326		-0.633		0.287		-0.198		-0.447
Age×distribution		0.075		0.139		0.055		0.557		0.714**		-0.076		0.026		-0.111
Age×VAS		-0.225		0.127		-0.477		0.156		-0.068		0.208		0.182		0.350
Gender×duration		-0.158		-0.005		-0.135		0.184		-0.105		-0.013		0.106		0.018
Gender×distribution		-0.064		0.191		-0.194		0.005		-0.138		-0.126		-0.046		-0.167
Gender×VAS		-0.211		-0.254		-0.464		0.038		0.138		0.094		-0.005		-0.447
R^2^ change	0.518**	0.044	0.438**	0.034	0.242**	0.082	0.110*	0.053	0.132*	0.100	0.758**	0.017	0.618**	0.008	0.734**	0.026
F change	21.984	1.531	15.297	0.986	6.272	1.865	2.434	0.978	2.993	2.006	61.499	1.155	31.703	0.346	54.104	1.640

Note:

* P <0. 05,

** P<0. 01.

^a^ Men = 0, Women = 1.

^b^ In years.

^C^ cover = 0 uncover and both = 1.

All the coefficients in the table are standardized regression coefficients

## Discussion

In recent years, both the importance of clinical symptoms and QoL have been recognized for the assessment of therapeutic effects. Doctors often define skin disease severity based on symptoms and the area of the skin lesions, but patients pay more attention to impaired activities of daily living. SF-36 has been proven useful in estimating the burdens of different diseases. However, the dermatology-specific QOL instrument (DLQI) is very useful for the assessment of relevant skin-specific features [10–12]. Combination of the DLQI with the generic SF-36 tool may give further insight into the burden experienced by PCA patients. The combination provides a more comprehensive assessment from different perspectives.

To the best of our knowledge, this is the first study to investigate QoL in PCA patients. The results of this study showed that PCA not only is a physical disorder but also diversely affects QoL. The mean DLQI of 9.05 in our patients with PCA is worse than that reported for cutaneous lupus erythematosus (6.5), Behcet’s syndrome (5.7), alopecia (8.3), and Darier’s disease (5.9), but better than that for pemphigus vulgaris (10.0), epidermolysis bullosa (12.1) and burns (17.7)[13–20]. In this study, we describe a strong impact of PCA on patients’ QoL both for dermatology-specific (using the DLQI) and for GH (using the SF-36) aspects. The mean total DLQI score was 9.05, indicating a moderate impairment in QoL, with the highest score found for symptoms and feelings, daily activities and personal relationships and the lowest score found for work and school and treatment. Our data suggest that PCA patients exhibit more psychological effects of the disease on QoL, as demonstrated by lower scores in social and emotional domains in the SF-36 tests. It is not difficult to imagine the psychosocial impairment of PCA. Consequently, it is important to realize that further investigations are needed to assess how psychological and social factors may influence the disease state in the PCA population.

As indicated by multiple regression analysis, the variance in DLQI outcome measures could be mainly accounted for by age, gender, distribution patterns of PCA and pruritus severity. Our results suggest that psychological symptoms are more severe in the young PCA patients than the older population. The young patients had higher DLQI scores and a lower RE score, which indicate that young patients experienced greater impairment of their QoL. Younger patients are usually more self-conscious and more socially active. Their relationship with others, self-image, and self-esteem can cause depression and emotional distress. The observation that the impact of PCA decreases with increasing age has also been demonstrated in other studies [21]. Because of old age, old patients usually had longer disease duration and may better cope with the disease. Studies performed by Zachariae et al. and Kanikowska et al showed that both genders reported a similar degree of impairment in their QoL [22–23]. However, our current observations indicate that women were more likely than men to report impairment in QoL. This finding is similar to those of previous studies carried out by Gelfand et al [24] and could be explained by females usually caring more about their self-appearance and social relationships than males. Females may also feel less attractive and are more concerned about lower chances of getting married. Therefore, these findings suggest that the QoL assessment plays a greater role in females than in males when assessing the severity of PCA.

Pruritus is an uncomfortable subjective sensation. Although it is difficult to define, it is obvious that it can be annoying. Studies suggest that pruritus may lead to sleep disorders and psychological problems, thereby affecting daily life activity and QoL. The study by Wright A et al showed a significant relationship between pruritus severity and skin-related QoL[25]. Pruritus has been noted as a clinical feature of PCA. Similarly, in the present study, pruritus was correlated with the QoL. The effect of pruritus was significant in both the models for DLQI and SF-36 scores. Because of its significant impact on QOL, pruritus management is an important component of PCA management. In our study, we observed a reverse association between the functioning component of QoL and localization on the uncover area. Patients of PCA with lesion in uncover area had worse functional QoL. The association between impaired QoL and anatomical sites has already been demonstrated [4]. These findings suggest that anatomical locations should be incorporated into a severity scoring system as weight-assigned variables. As shown in our studies, it would be expected that localization of dermatological conditions on visible parts of the body, such as the face and hands, should impair QoL more than localization on hidden parts.

Although PCA patients with a longer duration may have lower Qol, in our study, they did not impact the DLQI and SF-36. In the multifactorial model, duration of PCA was also not an independent predictor of either the DLQI or SF-36 score.

The current study has some limitations. First, all subjects were managed by dermatologists in one city. Enrolled participants were from primary dermatology clinics and are therefore not representative of the general PCA population; this potentially could have influenced the DLQI score. Second, the study was cross-sectional and did not discriminate between those who had initiated treatment and those who had not. To fully explore the relationship between disease activity and QoL, future studies on the changes in QoL as disease activity changes are warranted. In addition, the study is limited by the small study population. A larger patient population needs to be studied to further assess QoL in patients with PCA, specifically to better compare PCA with other diseases.

## Conclusions

In conclusion, the present study suggests that patients with PCA are highly affected in the functional and emotional aspects of QoL. Several clinical features, such as age, gender and distribution patterns, can also have important effects on QoL. Therefore, these clinical features should be considered when physicians start to frame a clinical interview form for PCA in their clinical practices.
